# Reduced suicidality after electroconvulsive therapy is linked to increased frontal brain activity in depressed patients: a resting-state fMRI study

**DOI:** 10.3389/fpsyt.2023.1224914

**Published:** 2023-07-12

**Authors:** Xue Wang, Han Wu, Dan Wang, Wei Wang, Wen Wang, Wen-qing Jin, Jiong Luo, Wei Jiang, Yi-lang Tang, Yan-ping Ren, Chun-lin Yang, Xin Ma, Rena Li

**Affiliations:** ^1^Beijing Key Laboratory of Mental Disorders, National Clinical Research Center for Mental Disorders & National Center for Mental Disorders, Beijing Anding Hospital, Capital Medical University, Beijing, China; ^2^Advanced Innovation Center for Human Brain Protection, Capital Medical University, Beijing, China; ^3^Department of Psychiatry and Behavioral Sciences, Emory University School of Medicine, Atlanta, GA, United States; ^4^Mental Health Service Line, Joseph Maxwell Cleland Atlanta VA Medical Center, Decatur, GA, United States; ^5^Beijing Institution of Mental Health Care, Beijing, China

**Keywords:** electroconvulsive therapy, suicidality, depressed patients, functional MRI, frontal regions

## Abstract

**Objective:**

Suicidality is commonly observed in patients with depressive episodes, and electroconvulsive therapy (ECT) has been found to be effective in treating these patients. However, the role of ECT in suicidality remains unclear. This study used resting-state functional magnetic resonance imaging (rs-fMRI) to explore the changes in brain function before and after ECT in depressed patients with suicidality.

**Methods:**

In total, 26 depressed patients with suicidality underwent rs-fMRI at baseline and after 8–12 sessions of ECT. In addition, 32 healthy controls (HCs) matched for age, gender, and educational level underwent rs-fMRI once. The amplitude of low-frequency fluctuations (ALFF), the fractional amplitude of low-frequency fluctuations (fALFF), and regional homogeneity (ReHo) were measured to evaluate whole brain function. Differences between the groups and time points (before and after ECT) were compared. Clinical symptoms were assessed using the 17-item Hamilton Depression Scale (HAMD-17) and Beck Scale for Suicide Ideation (BSSI).

**Results:**

At baseline, patients exhibited decreased ALFF in the right postcentral and precentral gyrus and decreased fALFF in the right supramarginal and postcentral gyrus, left superior frontal gyrus (SFG), as well as the superior and middle temporal gyrus compared to HCs. Patients also had lower ReHo in the left amygdala, anterior cingulate, and postcentral gyrus, and in the right thalamus, insula, and postcentral gyrus. They also exhibited higher ALFF in the bilateral temporal gyrus and insula as well as higher fALFF in the cerebellum. Following ECT, fALFF in the left SFG and orbital frontal cortex (OFC) significantly increased and was inversely correlated with the reduction of BSSI scores (*r* = −0.416, *p* = 0.048), whereas no correlation was found with changes in HAMD-17scores.

**Conclusion:**

Our findings suggest that the left SFG and OFC may play a key role in the mechanism of ECT for suicidality. The decrease of fALFF in the left SFG and OFC may represent a potential mechanism through which ECT effectively treats suicidality in depressed patients.

## 1. Introduction

Suicide is a major global public health concern, leading to 800,000 deaths annually. Depression accounts for approximately half to two-thirds of all suicides (1, 2), which suggests that patients with depression have the highest risk of suicide. Suicidality may have distinct mechanisms and risk factors compared to other depressive symptoms (3). Electroconvulsive therapy (ECT) has been shown to be effective in treating many severe mental illnesses, especially depressive episodes, and bringing about rapid improvements in severe clinical symptoms, especially suicidality (4, 5). Multiple reports have confirmed the efficacy of ECT in significantly reducing suicidality, including suicide ideation (SI) and suicide attempts (SA) (6, 7), in patients with both major depressive disorder (MDD) and bipolar disorder (BD). However, the underlying mechanism of the effects of ECT on suicidality remains poorly understood.

One possible explanation for the effectiveness of ECT in reducing suicidality is its impact on brain function in regions associated with mood regulation and behavior control. Resting-state functional magnetic resonance imaging (rs-fMRI) is a widely used imaging technique in psychiatric disorders, including depression, as it enables the evaluation of brain structure, activity, and blood flow. Measures such as the amplitude of low-frequency fluctuations (ALFF), fractional amplitude of low-frequency fluctuations (fALFF), and regional homogeneity (ReHo) are often utilized to assess spontaneous brain activity by reflecting the intensity, variability, and synchronization of neuronal activity.

A few previous studies have investigated changes in brain structures and functions associated with ECT for suicidality in patients with depression. One study focusing on adolescent patients with depression and SI (8) showed that the ALFF and fALFF values in the right precentral gyrus reduced significantly after ECT, compared to baseline. This reduction was inversely correlated with changes in the scores of the Beck Scale for Suicide Ideation (BSSI) after ECT. Another study, also involving adolescents with depression and SI, utilized rs-fMRI (9, 10) and suggested that the effects of ECT were associated with an increase in ALFF in the right middle occipital gyrus, as well as a decrease in ALFF in several brain regions, including the right inferior frontal gyrus (especially the opercular part), right frontal middle gyrus, and left temporal pole. Additionally, the ReHo increased in the left inferior temporal gyrus and right middle occipital gyrus after ECT. Moreover, a structural MRI study suggested that the gray matter volume increased in the right superior temporal gyrus and superior frontal gyrus after ECT in adolescent depressed patients with SI (9, 10). Although the above findings are interesting, it is important to note that there are inconsistencies among the different studies and a lack of research on the mechanism of ECT in young depressed patients with suicidality.

Our study aimed to clarify the mechanism underlying the effectiveness of ECT for suicidality in young patients experiencing depressive episodes. We compared whole-brain measures of ALFF, fALFF, and ReHo before and after ECT in depressed patients with suicidality. We hypothesized that ECT would reduce SI and increase brain activity in specific frontal regions in depressed patients. The findings of our study will contribute to a deeper understanding of the pathological mechanisms through which ECT exerts its effects on suicidality.

## 2. Materials and methods

### 2.1. Patients and healthy controls

Patients were recruited from Beijing Anding Hospital, Capital Medical University, Beijing, China. Patients aged 16–45 years who were right-handed and who met the diagnostic criteria for depressive episodes, as outlined in the Diagnostic and Statistical Manual of Mental Disorders, 5th Edition (DSM-5) (11), were included in the study. Participants were required to have a score of 17 or higher on the 17-item Hamilton Rating Scale For Depression (HAMD-17) (12) for inclusion in the study. Furthermore, individuals were included if they showed recent suicidal symptoms, including SI and SA, within the 2 weeks preceding the study. The decision to administer ECT was made by the treating psychiatrists based on the severity of depressive symptoms, the presence of SI, and the patient’s preferences.

The study excluded individuals with a history of schizophrenia or other mental disorders (e.g., intellectual disability), neurodegenerative or neurological disorders (Alzheimer’s disease, epilepsy, severe brain injury, etc.), current drug or alcohol abuse, MRI contraindications, allergies to muscle relaxants or anesthetics, pregnancy, or recent use of ECT or other neuromodulation therapies such as transcranial electrical stimulation within 90 days preceding the study.

Individuals who matched the patients in terms of sex, age, and educational level were recruited to serve as healthy controls (HCs). The HCs had no history of psychiatric disease or a family history of suicide or psychiatric illness. The same exclusion criteria applied to the HCs as those for the depressed patients.

All participants or their family members provided written informed consent before participating in the study. The study was approved by the Ethics Committee of Beijing Anding Hospital, Capital Medical University.

### 2.2. Assessment of clinical symptoms and cognitive function

Clinical symptoms were assessed using the HAMD-17 and suicidality was evaluated using the BSSI, a self-rating scale of 19 items designed to assess recent suicidal thoughts, plans, and behaviors (13). The BSSI can estimate the probability of suicidal attempts based on the scores of items 6–19, with higher scores indicating a higher suicide risk. The BSSI score ranges from 0 to 100.

In this study, primary treatment response was defined as a BSSI score of 0 after ECT treatment.

### 2.3. ECT procedure

ECT was administered using the Thymatron system (Somatics Thymatron®, Venice, FL, United States) at the ECT center in Beijing Anding Hospital. Bifrontal electrode placement sites were used for the ECT procedure. The half-age method was used to determine the stimulus dosage (14). Before the induction of anesthesia, all patients received intravenous administration of atropine (0.5 mg). Muscle relaxation was achieved by intravenous administration of succinylcholine (0.4 mg/kg for women, 0.5 mg/kg for men). Anesthesia was induced using intravenous propofol (1.5 mg/kg).

Each patient underwent 8–12 sessions of ECT treatment based on their treatment response. The treatment sessions were scheduled on Days 1, 2, 3, 4, 6, 8, 10, 12, and 14, with additional sessions on Days 16, 18, and 20, if deemed necessary.

### 2.4. Magnetic resonance imaging

The MR images were obtained at the Department of Medical Imaging, Beijing Anding Hospital, using a 3 T scanner with a standard 64-channel head–neck coil (MAGNETOM Prisma, Siemens Healthcare, Erlangen, Germany). The echo-planar imaging pulse sequence parameters were as follows: repetition time (TR) = 3,000 ms, echo time (TE) = 30 ms, field of view (FOV) = 216 × 216 mm^2^, matrix = 64 × 64, flip angle (FA) = 90°, slice number = 47, slice thickness = 3 mm, voxel size = 3 × 3 × 3 mm^3^. A T1-weighted structural image was acquired using a 3D magnetization-prepared, rapid acquisition gradient echo sequence with the following parameters: TR = 2,530 ms, TE = 1.85 ms, FOV = 256 × 256 mm^2^, FA = 90°, voxel size = 1 × 1 × 1 mm^3^, slice thickness = 1.0 mm, matrix = 256 × 256. All image acquisitions were performed by the same chief radiologist.

All depressed patients with suicidality underwent MRI at baseline and 24 h after ECT, whereas the HCs underwent MRI only once.

### 2.5. Image analysis

In the MATLAB 2020a platform, rs-fMRI data were preprocessed using the DPABI V7.0 toolbox (15) using the following steps:

(1) The raw image format was converted from DICOM to NIFTI format; (2) The first 10 time points from the original image were removed to ensure signal stability; (3) Layer time correction was performed; (4) Head movement was corrected at the individual level using the Friston 24 model, and subjects with significant head movements (Mean FD_Jenkinson >0.2 mm) were excluded; (5) Spatial standardization was performed to map the data to the standard space of the Montreal Neurology Institute. The voxel size was resampled to 3 mm × 3 mm × 3 mm; (6) Gaussian smoothing kernel was used for image smoothing to refine the signal-to-noise ratio; (7) Linear drift was removed to reduce the impact of physiological low-frequency noise; (8) A filter was applied to maintain frequencies in the range of 0.01–0.1 Hz; (9) *Post hoc* Z-standardization was performed on all RS-fMRI related indicators to make the data more comparable. This study did not perform regression processing on global signals.

After preprocessing the RS-fMRI data, the DPARSF V5.4 toolkit was used for ReHo analysis. The time series similarity between the voxel and its neighboring voxels was evaluated using Kendall’s Coefficients of Concordance (KCC). The KCC value ranged from 0 to 1, with a larger KCC value indicating better consistency and a lower value indicating lower consistency. Whole-brain ReHo images were generated by computing the KCC values for all brain voxels.

The DPARSF V5.4 toolkit was also used to perform Fast Fourier Transform on the time series of blood-oxygen-level-dependent signals to calculate the power spectrum of the functional signals across the whole brain. The average square root of the power spectrum was used to calculate the ALFF value for each voxel. The fALFF values were obtained by computing the ratio of the power within a specific frequency band to the power across the entire range of all detected frequency bands. The values of ALFF and fALFF were computed before and after ECT for patients and HCs.

### 2.6. Sample size calculation

To calculate the sample size for the study, we assumed an effect size of 0.57–0.68 for the efficacy of ECT in depressed patients, a power of 0.80, and a type 1 error rate of 0.05. The minimum sample size calculated using the power analysis and sample size software was 27. Considering a potential loss of 30% during the study because of factors such as MRI scanning and ECT treatment in depressed patients, we decided to include a total of 35 patients and 35 matched HCs.

### 2.7. Statistical analysis

Statistical analysis was conducted using the SPSS software (version 26.0, SPSS Inc., Armonk, NY, United States). To compare the demographic characteristics between the depressed patients and HCs, *χ*^2^ tests were used for gender and marital status, whereas independent sample t-tests were used for age and education level. Paired sample t-tests were performed to compare the differences in the scores of HAMD-17 and BSSI before and after ECT treatment in the patients.

The Statistical Analysis module in the DPABI V7.0 toolbox was used to analyze the ALFF and fALFF values and ReHo. Independent sample *t*-tests were adopted to assess the differences in ReHo and ALFF/fALFF values between HCs and patients. Paired *t*-tests were used to compare ReHo and ALFF/fALFF values before and after ECT treatment in patients. Head motion parameters (Mean FD_Jenkinson) were included as covariates in the analysis. The significance level for this study was set at *p* < 0.05 (two-tailed), with Gaussian random field (GRF) correction applied. The ROI Signal Extractor module in the DPABI V7.0 toolbox was used to extract statistically significant brain regions. Correlation coefficients among ReHo, ALFF/fALFF, HAMD-17 scores, and BSSI scores were calculated using the Pearson test. The significance level was set at *p* < 0.05 (two-tailed). Alterations in HAMD-17 (ΔHAMD) and BSSI (ΔBSSI) after ECT treatment compared to baseline were calculated using the equations:

ΔHAMD=post−HAMD−pre−HAMD

ΔBSSI=post−BSSI−pre−BSSI

Alterations in ReHo (ΔReHo) and ALFF/fALFF (ΔALFF/fALFF) were calculated using the equations:

ΔReHo=post−ReHo−pre−ReHo

ΔALFF/fALFF=post−ALFF/fALFF−pre−ALFF/fALFF

## 3. Results

### 3.1. Clinical outcomes

In total, 35 depressed patients and 35 HCs were initially enrolled in this study. However, nine depressed patients and three HCs were excluded owing to their incomplete MRI recordings or MR image that did not meet the criteria for analysis. Finally, 26 depressed patients (19 with MDD and 7 with BD) and 32 HCs completed the entire study protocol. At baseline, no significant differences were observed in age, gender, marital status, or education level between the depressed patients with suicidality and the HC group (*p* > 0.05). The mean duration of illness in the patients was 5.97 ± 5.92 years. All depressed patients reported either recent SAs (*N* = 10) or current SIs (*N* = 16). All patients were taking psychotropic medications, with 21 taking antidepressants, 9 taking mood stabilizers, and 14 taking antipsychotics. During the ECT treatment, 5 patients had their antidepressants changed, with 3 patients switching to venlafaxine and 2 switching to sertraline. Further details are provided in Table 1.

**Table 1:** Comparison of demographic characteristics between 32 healthy controls (HCs) and 26 depressed patients with suicidal ideation (SI) or recent suicide attempts (SAs).

Characteristic	HCs (*n* = 32)	Patients (*n* = 26)	*t/χ^2^*	*p*
Age (years)	29.63 ± 7.53	27.73 ± 7.59	0.949	0.347
Gender (*n*, %)			3.205	0.073
Male	10 (31.3)	3 (11.5)		
Female	22 (68.8)	23 (88.5)		
Marriage (*n*, %)			–	0.592^a^
Unmarried	17 (53.1)	15 (57.7)		
Married	15 (46.9)	10 (38.5)		
Divorced	0 (0.0)	1 (3.8)		
Education (*n*, %)			–	0.136^a^
Secondary school	4 (12.5)	7 (26.9)		
University	22 (68.8)	18 (69.2)		
Master’s degree or higher	6 (18.8)	1 (3.8)		
Duration of illness (years)	–	5.97 ± 5.92	–	–
Medications (*n*, %)				
Antidepressants		21 (80.7)		
Sertraline		4 (15.4)		
Mirtazapine		1 (3.8)		
Venlafaxine		3 (11.5)		
Escitalopram		8 (30.8)		
Duloxetine		3 (11.5)		
Fluoxetine		2 (7.7)		
Antipsychotics		14 (53.7)		
Quetiapine		11 (42.3)		
Lurasidone		1 (3.8)		
Aripiprazole		1 (3.8)		
Olanzapine		1 (3.8)		
Mood stabilizers		9 (34.5)		
Lamotrigine		2 (7.7)		
Lithium		7 (26.9)		
Suicidal symptoms (*n*, %)				–
SI		16 (61.5)		
SA		10 (38.5)		
Diagnosis (*n*, %)				–
MDD		19 (73.1)		
BD		7 (26.9)		

*p < 0.05.

a Fisher exact probability.

MDD, major depressive disorder; BD, bipolar disorder.

After completing 8–12 sessions of ECT treatment, 23 out of 26 patients (3 women and 20 men) reported a resolution of suicidal risk, and 3 patients (all women) continued to endorse suicidality.

Among the ECT-responsive patients, both HAMD-17 and BSSI scores significantly decreased compared to baseline (*p* < 0.001). However, in the non-ECT-responsive patients, no significant reductions were observed in HAMD-17 or BSSI scores compared to baseline (*p* > 0.05; Table 2).

**Table 2:** Comparison of HAMD-17/BSSI scores before and after ECT in depressed patients.

Characteristic	ECT-responsive (*n* = 23)		*t*	*p*	Non-ECT-responsive (*n* = 3)		*t*	*p*
	Pre-ECT	Post-ECT			Pre-ECT	Post-ECT		
HAMD-17	28.91 ± 5.95	11.43 ± 3.12	12.894	<0.001^*^	33.33 ± 3.51	26.67 ± 4.16	2.561	0.125
Anxiety somatization factor	7.26 ± 2.22	4.30 ± 1.02	5.998	<0.001^*^	7.67 ± 2.08	7.33 ± 1.53	0.500	0.667
Weight factor	1.17 ± 0.65	0.13 ± 0.34	7.091	<0.001^*^	1.00 ± 1.00	1.00 ± 0000	0.000	1.000
Cognitive impairment factor	6.74 ± 2.16	2.57 ± 1.27	8.194	<0.001^*^	7.67 ± 0.58	0.67 ± 2.08	7.000	0.020^*^
Retardation factor	8.83 ± 1.53	3.26 ± 1.25	13.371	<0.001^*^	10.33 ± 0.58	7.33 ± 1.15	3.000	0.095
Sleep disorder factor	3.96 ± 1.40	0.65 ± 0.57	10.223	<0.001^*^	5.33 ± 0.58	2.67 ± 0.58	4.000	0.057
BSSI	43.74 ± 20.83	0.00	10.070	<0.001^*^	68.69 ± 22.95	45.45 ± 12.12	3.190	0.086

^*^p < 0.05.

HAMD-17: 17-item Hamilton Depression Scale; BSSI: Beck Scale for Suicide Ideation; ECT: electroconvulsive therapy.

The mean age of the non-ECT-responsive patients was 19.00 ± 2.65 years, and the mean duration of illness was 2.33 ± 1.15 years. These 3 patients had not received ECT in the past, and were taking different medications: one patient was taking quetiapine (25 mg/day) combined with escitalopram (10 mg/day) for about 1 month, another patient was taking duloxetine (40 mg/day), and the third patient was taking lamotrigine (25 mg/day). Despite taking medications, their symptoms failed to improve, and suicidality persisted before ECT. The average seizure duration of all participants who received ECT was 30.76 ± 8.86 s. The average seizure durations of the 23 responsive patients and the 3 responsive patients were 31.40 ± 9.17 s and 25.86 ± 3.82 s, respectively.

### 3.2. Baseline comparisons of ReHo and ALFF/fALFF between depressed patients and HCs

**Figure 1:**
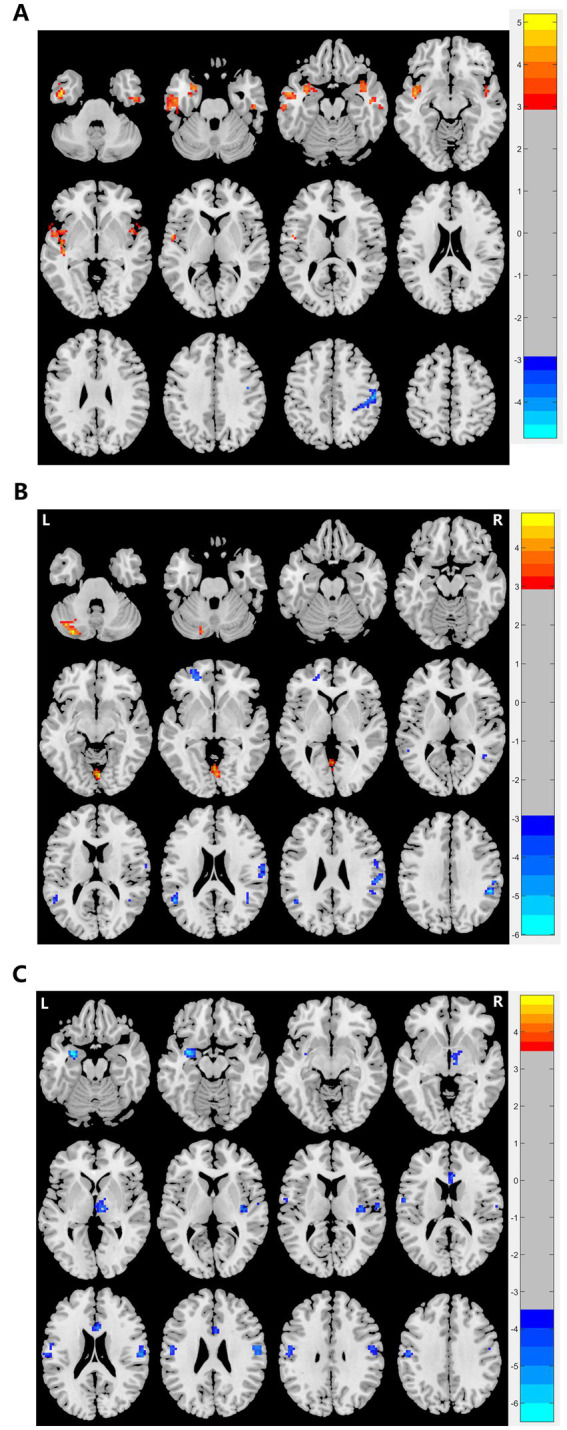
Graphs show significant differences in brain regions in **(A)** ALFF values between patients and HCs at baseline. The blue region indicates a decreased ALFF value in the post/precentral gyrus compared to HCs. Red regions indicate increased ALFF values in the left and right temporal lobe and insula compared to HCs. GRF correction, voxel *p* < 0.005, cluster *p* < 0.05. **(B)** ReHo between patients and HCs at baseline. Red regions indicate increased ReHo in the left amygdala, right thalamus, right and left postcentral, and left and right ACC compared to HCs. GRF correction, voxel *p* < 0.005, cluster *p* < 0.05. **(C)** fALFF values between patients and HCs at baseline. The blue region indicates a decreased fALFF value in the left superior frontal gyrus and left superior and middle temporal gyrus compared to HCs. Red regions indicate increased fALFF values in the lingual and cerebellum compared to HCs. GRF correction, voxel *p* < 0.005, cluster *p* < 0.05. ALFF, amplitude of low-frequency fluctuation, fALFF, fractional amplitude of low-frequency fluctuation, ReHo: regional homogeneity, HCs, healthy controls, ACC, anterior cingulate gyrus, GRF, Gaussian random field.

We observed significant differences in the ReHo and ALFF/fALFF values between depressed patients with suicidality and HCs (Table 3 and Figure 1). Compared to HCs, patients had lower ALFF in the right precentral and postcentral gyrus and decreased fALFF in the right supramarginal and postcentral gyrus, left superior frontal gyrus, and left superior and middle temporal gyrus. In addition, they had lower ReHo in the left amygdala, right thalamus, insula, postcentral gyrus, left anterior cingulate, and left postcentral gyrus. Patients also showed increased ALFF values in the bilateral temporal gyrus and insula, and higher fALFF values in the cerebellum.

**Table 3:** Significant differences in ALFF/fALFF and ReHo between patients with MDD and HCs.

	Brain regions	Hemisphere	Voxel size	Peak *t* value	MNI coordinates		
Decreased							
ALFF	Post-central and pre-central	R	83	−4.3895	51	−21	42
fALFF	Supramarginal and post-central	R	196	−6.0142	51	−39	30
fALFF	Frontal_sup	L	48	−4.8359	−21	48	−6
fALFF	Temporal_sup and mid	L	47	−5.0501	−51	−48	18
ReHo	Amygdala	L	51	−6.5025	−30	−3	−18
ReHo	Thalamus	R	43	−4.6189	12	−15	3
ReHo	Insula	R	31	−5.4928	36	−15	9
ReHo	Postcentral	R	82	−5.4352	60	−15	21
ReHo	ACC_sup	L&R	55	−5.1997	3	27	18
ReHo	Post-central	L	75	−4.9741	−54	−15	36
Increased							
ALFF	Temporal and insula	L	477	5.1970	−48	3	−42
ALFF	Temporal	R	146	4.8236	39	3	−45
ALFF	Insula and temporal	R	80	4.0713	48	9	−6
fALFF	Lingual	L&R	70	4.8111	3	−75	−9
fALFF	Cerebellum	L	67	4.8948	−30	−78	−33

ALFF, amplitude of low-frequency fluctuations; fALFF, fractional amplitude of low-frequency fluctuations; ReHo, regional homogeneity; ACC, anterior cingulate gyrus; MNI, Montreal Neurological Institute; MDD, major depressive disorder; HCs, healthy controls.

GRF correction, voxel *p* < 0.005, cluster *p* < 0.05.

**Figure 2:**
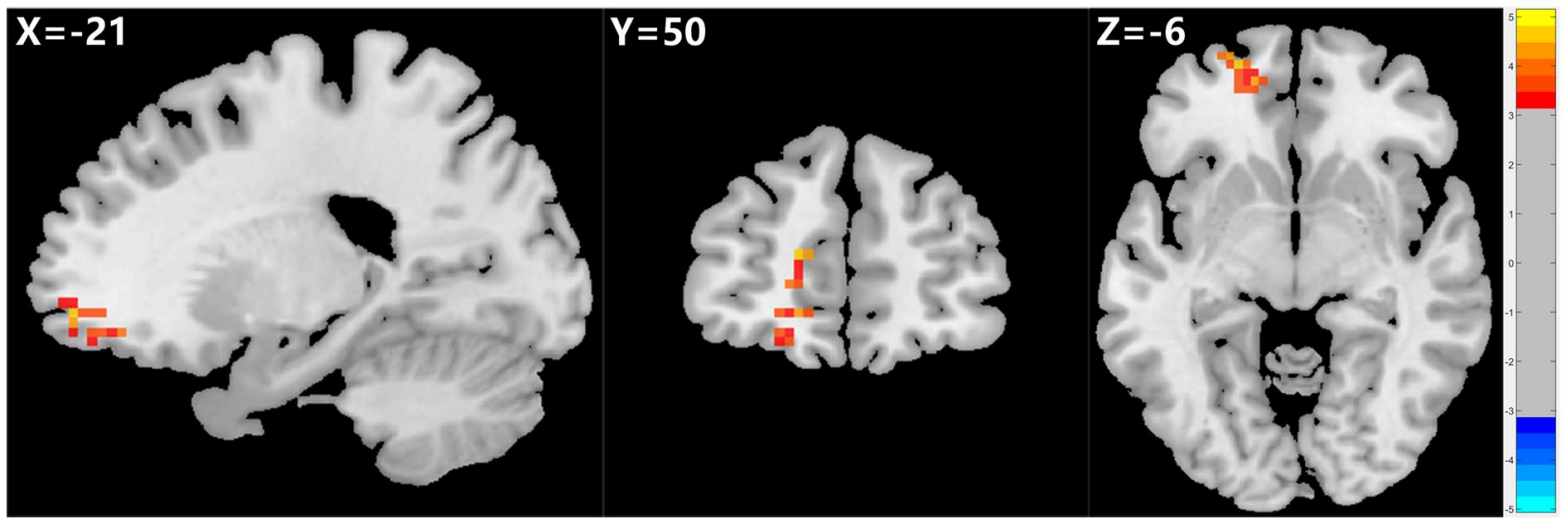
Graph shows a comparison of fALFF in the left SFG and OFC before and after ECT in depressed patients with suicidality. A significant increase in fALFF was observed in the left SFG and OFC after ECT compared to pre-ECT values. GRF correction, voxel *p* < 0.005, cluster *p* < 0.05. fALFF, fractional amplitude of low-frequency fluctuation, SFG, superior frontal gyrus, OFC, orbital frontal cortex, ECT, electroconvulsive therapy, GRF, Gaussian random field.

### 3.3. Changes in ReHo and ALFF/fALFF values in depressed patients with suicidality before and after ECT

The 23 patients who responded to ECT exhibited a marked rise in fALFF values in the left SFG and OFC after ECT, unlike patients who did not respond to ECT. However, ReHo and ALFF values did not show any significant changes before and after ECT (see Table 4; Figure 2).

**Table 4:** Significant differences in fALFF of depressed patients before and after ECT treatment.

	Brain regions	Hemisphere	Voxel size	Peak t value	MNI coordinates		
Increased							
fALFF	Frontal_Sup&OFC_ant	L	68	4.7677	−21	57	−6

fALFF, fractional amplitude of low-frequency fluctuations; MNI, Montreal Neurological Institute; ECT, electroconvulsive therapy.

GRF correction, voxel *p* < 0.005, cluster *p* < 0.05.

### 3.4. Correlation analysis

**Figure 3:**
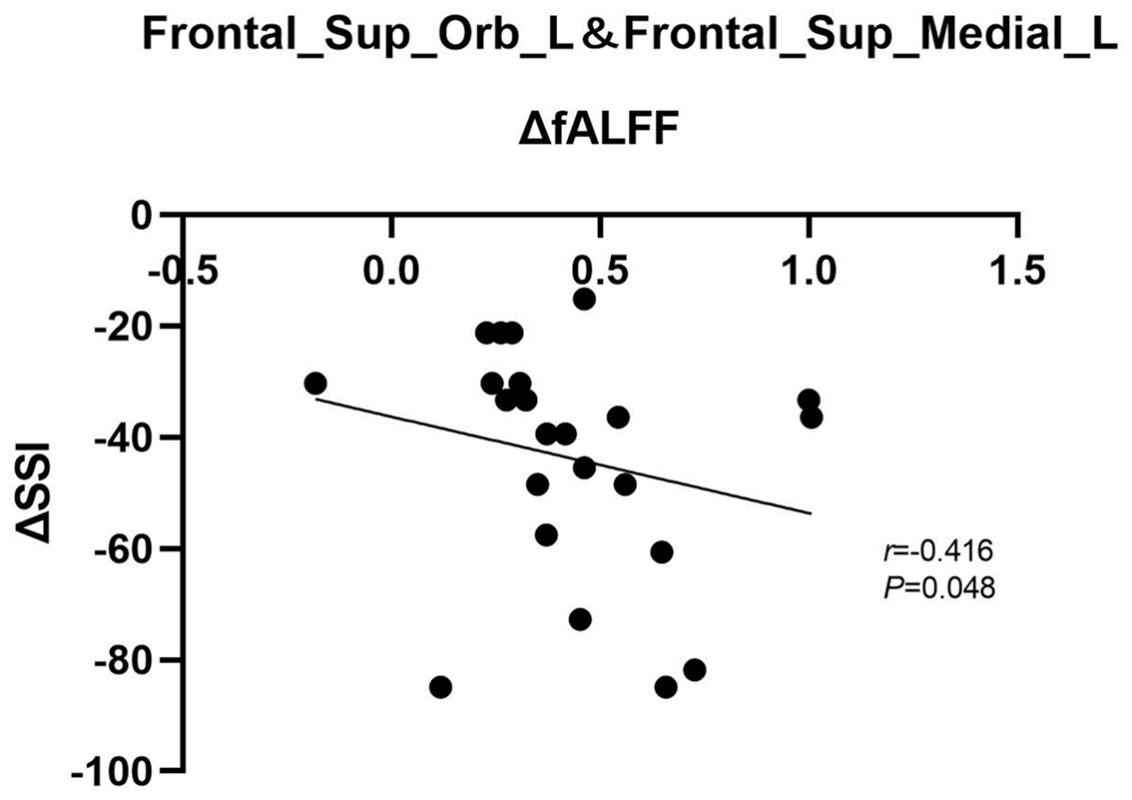
Graph shows a negative correlation between ΔBSSI and ΔfALFF in the left SFG and OFC after ECT. BSSI: Beck scale for suicide ideation, fALFF, fractional amplitude of low-frequency fluctuation, SFG, superior frontal gyrus, OFC, orbital frontal cortex, ECT, electroconvulsive therapy.

In the patient group, changes in fALFF (ΔfALFF) in the left OFC and SFG were adversely correlated with the changes in BSSI scores (ΔBSSI) before and after ECT (*r* = −0.416, *p* = 0.048). No significant associations were found between ΔfALFF in the left SFG and OFC and ΔHAMD-17 scores or its subscale scores (all *p* > 0.05) (see Figure 3).

## 4. Discussion

Based on a clinical sample of individuals with depression and suicidality, we investigated how ECT affects brain function and clinical variables in patients with suicidality. We observed significant improvements in both depressive symptoms and suicidality after ECT, which is consistent with previous reports (16). In addition, the fALFF value in the SFG was significantly lower in patients than in HCs. However, in depressed patients who responded to ECT, the fALFF values in the left OFC and SFG increased significantly after ECT, whereas non-responders did not show such changes. Furthermore, we found that the changes in fALFF values in the left SFG and OFC were negatively correlated with the changes in BSSI scores from baseline, but not with the changes in HAMD-17 scores. The influence of anesthesia on brain activity after ECT should be considered. Fortunately, the anesthetic used (propofol) has a relatively short half-life of approximately 1.8–8.3 min and is excreted from the body within 40 min (17). All depressed patients with suicidality underwent MRI 24 h after ECT, indicating that over 99% of propofol had been excreted from their system, minimizing any potential effects on brain activity.

This study is among the first few studies to investigate the impact of ECT on brain function in young depressed patients experiencing suicidality, using the rs-fMRI technique. To the best of our knowledge, only two previous studies have examined ECT-induced changes in brain activity among depressed adolescents with SI (8–10). One study showed that ECT was associated with a decrease in ALFF/fALFF in several regions, such as the precentral gyrus, temporal pole, and occipital gyrus, which were associated with improvements in depressive and suicidal symptoms (8). Another study (9) found that adolescents with MDD displayed changes in brain activity in several brain areas, such as higher ALFF and ReHo in the right middle occipital gyrus, lower ALFF in the left inferior frontal gyrus and right frontal middle gyrus, left temporal pole and opercular part, and increased ReHo in the left inferior temporal gyrus. The authors found that the values of ALFF in the right middle occipital gyrus and left inferior temporal gyrus were negatively correlated with the HAMD and BSSI scores, suggesting the involvement of these regions in the underlying mechanism of both depressive symptoms and suicidality in adolescents. Our study also showed the involvement of some of these regions, in addition to the left OFC and SFG, which differed from previous findings. These regions may be involved in the mechanisms underlying the development of depression and suicidality in adolescents. These disparities may reflect the developmental differences between young and adolescents with depression. Previous studies have indicated that the cortical gray matter is thinner in depressed adults than in HCs, but such differences have not been observed in depressed adolescents (18). Further studies are required to examine the differences in brain activity among patients belonging to different age groups, to provide a basis for personalized ECT interventions for suicidality.

Previous studies have linked brain activity in the SFG, which is a part of the prefrontal cortex (PFC), and the severity of depressive symptoms. The PFC plays an important role in stress perception, emotion regulation, and working memory (19). Dysfunction in the SFG, a key region of the PFC, is associated with impaired stress perception and emotion regulation. Studies have shown that ECT can affect brain activity in the SFG, which may be associated with improvements in depressive symptoms (20). The SFG is a key node in the frontoparietal network (FPN), a cognitive control system responsible for cognitive control and coordination between brain networks (21). FPN dysfunction can impair cognitive control abilities and decision-making performance, which are characteristic neurocognitive features in depressed patients with suicidality. It has been found that abnormal connectivity within the FPN could differentiate patients with SA from those without SA (22). A study (23) found decreased connectivity in the middle frontal gyrus and superior parietal lobule in patients with MDD and SI compared to depressed individuals without SI. Another study (24) also found a significant correlation between decreased brain connectivity within the FPN and the severity of suicide ideation in depressed patients. SFG is a key brain area involved in both depression and suicidality. Our results suggest that alterations in brain activity in the SFG are associated with improvements in suicidality, suggesting that the SFG might have an important role in ECT treatment for suicidality. The OFC, another brain region involved in cognitive functions such as emotion regulation, decision-making, and risk avoidance, is also associated with suicidality (25, 26). Previous studies (27, 28) have linked structural and functional abnormalities in the OFC to suicide. For example, decreased volume in the medial and lateral OFC has been observed in young adults and adolescents with BD who have also had recent SAs (29). Emilie et al. found increased brain activation in the lateral and medial OFC when adult patients with MDD and SAs were asked to respond to angry faces (27). In addition, an increase in brain activation in the lateral and medial OFC was observed in adults with MDD and SAs when they were required to respond to winning a reward (27). Another study reported that patients with BD with SAs demonstrated increased activation in the lateral OFC when specifically asked to recall and control negative autobiographical memories (28). Moreover, in veterans with SI, a higher level of activation in the lateral OFC was observed in a response inhibition task during error trials (30). These findings suggest that the OFC is involved in various cognitive functions, such as emotion regulation, facial emotion cognition, risk decision-making, reward processing, and response inhibition, which may influence suicidality risks. Therefore, we postulate that ECT modulates OFC activity and improves emotional and cognitive functions in depressed patients, leading to improvements in suicidal symptoms.

We did not find a correlation between changes in ReHo, ALFF, and fALFF in brain regions and changes in HAMD scores after ECT. However, we found significant differences in brain activity in several brain regions between depressed patients with suicidality and HCs in various brain regions, including the right postcentral and precentral gyrus, right thalamus, bilateral temporal gyrus, left amygdala, insula, anterior cingulate, postcentral gyrus, and the cerebellum. These brain regions have been linked to symptoms of suicidality and depression (31, 32). Furthermore, some of these regions are also involved in cognitive function. For example, the temporal lobe and thalamus play a role in emotional regulation and cognitive function. Impairments in the temporal lobe may disrupt emotional regulation and increase the risk of suicide (33, 34). The insula, a deep part of the cortex, relays information and processes emotions, the autonomic system, and visceral movement (5, 35, 36). Studies have linked changes in the insula to suicidality (37). For example, lower insular volume and activity were observed in individuals with BD with SAs (38). The amygdala and anterior cingulate regions are also involved in emotion regulation, and deficiencies in these areas can induce depression. The cerebellum is not only related to balance and motor coordination but also plays a key role in cognitive and emotional processes, as well as the occurrence and development of depression and suicidality (39, 40). The brain regions involved in suicidality, depression, and cognition may show overlapping as well as distinct characteristics from each other. The lack of correlation between changes in brain activity and HAMD scores in our study may be attributed to the small sample size and insufficient sensitivity of brain activity measures. Further studies involving larger sample sizes and more precise assessments of brain activity are needed to explore the underlying brain dysfunctions in these regions associated with suicidality and depressive symptoms.

Our study has several limitations that should be acknowledged. First, our sample size was relatively small; studies with larger sample sizes are needed to replicate our findings in the future. Second, all our patients were taking antidepressants, and we did not have a control group of patients who were taking only antidepressants. Therefore, the potential effects of medications on brain activity and other measures cannot be ruled out. Finally, some studies have suggested potential differences in brain imaging patterns between patients with SAs and those with SI (41). Wagner et al. found that the ALFF in the hippocampus and thalamus could potentially distinguish SA from SI, as well as from patient controls and healthy controls, indicating that the abnormality of brain activity in depressed patients with SA might be more severe. Unfortunately, owing to the relatively small sample size in our study, we did not differentiate the effects of ECT on brain function between SI and SA. Further research is needed to investigate the variations in brain activity and treatment response to ECT between patients with SAs and SI.

## 5. Conclusion

Our study focused on depressed patients with suicidality, including SAs and SI. We measured the fALFF in different brain regions before and after ECT treatment. We found that ECT-responsive patients showed a significant increase in fALFF in the left SFG and OFC and that this change was correlated with a decrease in BSSI scores, but not with changes in HAMD-17 scores. Thus, ECT may effectively modulate brain function in the left SFG and OFC, consequently reducing suicidal symptoms among depressed patients.

## Data availability statement

The original contributions presented in the study are included in the article/supplementary materials, further inquiries can be directed to the corresponding authors.

## Ethics statement

The studies involving human participants were reviewed and approved by the Ethics Committee of Beijing Anding Hospital, Capital Medical University. Written informed consent to participate in this study was provided by the participants' legal guardian/next of kin.

## Author contributions

XW designed the study and wrote the first draft of the manuscript. HW administered neuropsychological and clinical measures, completed the analyses, assisted in writing, and editing of the manuscript. DW, WiW, WnW, and W-qJ recruited participants, administered neuropsychological, and clinical measures. JL helped design the study and recruited participants. WJ performed ECT. Y-lT assisted in writing and editing of the manuscript. Y-pR designed the study and assisted in writing and editing of the manuscript. C-lY administered the MRI data processing. XM and RL designed the study. All authors contributed to the article and approved the submitted version.

## Funding

The research conducted by RL was supported by the National Key Research and Development Program of China (no: 2020YFC2005300), and that conducted by Y-pR was supported by the Capital Health Research and Development of Special Projects (2018–3-1171).

## Conflict of interest

The authors declare that the research was conducted in the absence of any commercial or financial relationships that could be construed as a potential conflict of interest.

## Publisher’s note

All claims expressed in this article are solely those of the authors and do not necessarily represent those of their affiliated organizations, or those of the publisher, the editors and the reviewers. Any product that may be evaluated in this article, or claim that may be made by its manufacturer, is not guaranteed or endorsed by the publisher.
